# Severe Hyponatremia Due to Cisplatin-induced Syndrome of Inappropriate Secretion of Antidiuretic Hormone

**DOI:** 10.7759/cureus.5458

**Published:** 2019-08-22

**Authors:** Haisam Abid, Nadir Siddiqui, Rosana Gnanajothy

**Affiliations:** 1 Internal Medicine, Bassett Medical Center, Cooperstown, USA; 2 Oncology, Bassett Medical Center, Cooperstown, USA

**Keywords:** cisplatin, severe hyponatremia, siadh

## Abstract

We report a rare case of severe hyponatremia due to cisplatin-induced syndrome of inappropriate secretion of antidiuretic hormone (SIADH) in a 74-year-old male with a history of squamous cell carcinoma of the tongue who presented with a five days history of worsening confusion. He was found to have severe hyponatremia. His clinical and laboratory findings were consistent with hyponatremia due to SIADH. He was treated with fluid restriction and salt tablets after which his serum sodium markedly improved. Drugs including diuretics, antidepressants, antipsychotics are well known to cause hyponatremia. Cisplatin is a very common antineoplastic drug for solid tumors. Clinicians need to have prompt recognition of this rare side effect of cisplatin to prevent life-threatening hyponatremia.

## Introduction

Hyponatremia is a very common metabolic disturbance in patients with cancer. Pathophysiology differs, but most commonly it is related to syndrome of inappropriate secretion of antidiuretic hormone (SIADH), which results in increased re-absorption of free water. More common side effects of cisplatin include nephrotoxicity, anaphylaxis, and cytopenias. Hyponatremia is a rare complication associated with cisplatin. The mechanism by which it causes hyponatremia is thought to be due to either inducing SIADH or by renal salt wasting syndrome (RSWS) [1]. It is unclear why it occurs but it is believed that pharmacogenetics plays an essential role. We herein report a case of severe, rapidly developing hyponatremia after initiating cisplatin chemotherapy. Hyponatremia resolved after fluid restriction and salt tablets. His chemotherapy was switched to carboplatin and paclitaxel.

## Case presentation

Our patient is a 74-year-old male, with a 40 pack-year smoking history, with recently diagnosed stage II squamous cell carcinoma of the base to the tongue. According to the National Comprehensive Cancer Network (NCCN) guidelines, he was started on cisplatin. He presented to the emergency department five days after the initiation of cisplatin with worsening confusion and anxiety. He was also having some word-finding difficulty and was more forgetful. The patient denied seizures, syncope, chest pain, shortness of breath, weakness/numbness, diarrhea, polyuria, vomiting or thirst. There was no history of alcohol consumption and diuretic intake. On examination, the patient had normal vitals and euvolemic without any focal neurologic deficits. He was found to have a serum sodium of 115 mmol/L. Prior to starting chemotherapy, his serum sodium was 138 mmol/L and chloride was 102 mmol/L. He had normal kidney function. Other electrolytes were within the normal range. Serum osmolality was 244 mOsm/kg with urine osmolality of 422 mOsm/Kg and urine sodium of 68 mmol/L consistent with SIADH. Thyroid-stimulating hormone (TSH) level was 1.44 uIU/ml (normal range 0.34-3.0 uIU/ml) and serum cortisol was 17.9 ug/dl (normal range 3.0-27.0 ug/dl) which ruled out hypothyroidism and adrenal insufficiency. Chest imaging was within normal limit without any pulmonary metastasis. Based on the temporal relationship of his condition with chemotherapy, diagnosis of cisplatin-induced hyponatremia due to SIADH was made after ruling out other causes of hyponatremia.

His fluid intake was restricted to less than one liter per day and he was also started on salt tablets with goal of sodium correction of no more than 6 mmol/L in 24 hours. On this regimen, his sodium markedly improved upto 129 mmol/L and he was discharged home with fluid restriction and salt tablets. His chemotherapy was switched to carboplatin and paclitaxel and he was continued on radiation treatment as an out patient. His sodium steadily improved and went up to 140 mmol/L in three months duration. His salt tablets were steadily tapered down over one month and later discontinued.

## Discussion

Hyponatremia is the most common metabolic disturbance seen in cancer patients. There are multiple causes of hyponatremia in a cancer patient. Cisplatin was the first platinum-based chemotherapeutic agent that was identified to be effective against a wide variety of solid tumors [1]. Cisplatin is classically associated with nephrotoxicity and it usually causes kidney dysfunction with a decrease in glomerular filtration rate. Cisplatin is more frequently found to be associated with hyponatremia than carboplatin. In a patient with malignancy, hyponatremia is most likely due to increased free water intake due to SIADH, poor solute intake or metastatic disease leading to adrenal insufficiency, Cisplatin-induced hyponatremia is a very rare side effect and it can be due to either cisplatin-induced SIADH or RSWS [2].

In 1982, Levin et al. first described the case of cisplatin-induced hyponatremia in a patient with malignant thymoma. Subsequently, it has been reported in patients with ovarian cancer, non-small cell lung cancer, esophageal cancer, cervical neuroendocrine tumor, squamous cell carcinoma, pediatric osteosarcoma, and urothelial carcinoma [3]. To the best of our knowledge, it is the first case of cisplatin-induced hyponatremia in a patient with squamous cell carcinoma of the base of the tongue. Cisplatin can cause severe symptomatic hyponatremia resulting in seizures, coma, and even death. Renal tubular necrosis and impaired sodium reabsorption have been proposed as the possible mechanisms of cisplatin-induced hyponatremia [4]. Neurotoxicity related to cisplatin can also be a contributing factor causing hyponatremia due to SIADH [3].

Most of the case reports of cisplatin-induced hyponatremia have been reported in Japan or Korea. The increased incidence of cisplatin-induced hyponatremia in the East Asian population might be due to differences in pharmacogenetics as compared to other races [3]. Certain genetic profiles have been found which correlate with different toxicities of cisplatin such as ototoxicity and bone marrow suppression. Assessment of volume status in a patient is critical in differentiating the cause of cisplatin-induced hyponatremia: patient appears hypovolemic in RSWS and euvolemic in SIADH. Serum osmolality, urine osmolality, and sodium can be helpful in the diagnostic approach [2]. Other causes of euvolemic hyponatremia such as hypothyroidism and adrenal insufficiency should be ruled out, as in our patient [5]. Our patient was euvolemic on exam and lab work up was suggestive of hyponatremia due to SIADH.

There are some studies on animal models suggesting the role of antioxidants such as N-acetyl cysteine, vitamin E and C in renoprotection in patients of cisplatin. However, there is no clear-cut clinical data to support it. Cisplatin should be switched to carboplatin in patients with severe cisplatin-induced hyponatremia despite the effect that cisplatin has been proven to be superior to carboplatin in terms of antitumor effects in various cancers [4].

## Conclusions

Cisplatin-induced SIADH leading to hyponatremia is a rare side effect. Assessment of the volume status is critical in differentiating between SIADH and RSWS. Treatment options include fluid restriction and salt tablets. Alternative chemotherapy regimens should be considered.
